# The Treatment of Disease by Electric Currents

**Published:** 1899-02

**Authors:** 


The Treatment of Disease by Electric Currents. A Hand-Book of Plain Instructions for the General Practitioner. By S. H. Monell, M. D., Founder and Chief Instructor of the Brooklyn Post-Graduate School of Clinical Electro-Therapeutics and Roentgen Photography; Fellow of the New York Academy of Medicine, etc., etc. Published by William Beverly Harrison, 3 and 5 West Eighteenth St., N. Y. 1897. Pages, 1100. Price, cloth, $7.50, net. Postage, 50 cents.

A few years since, when the modern age of electricity began, extravagant hopes of its usefulness as a therapeutic agent were entertained, which as might have been anticipated have not as yet been realized. The pathologist, the chemist, the bacteriologist, the physiologist and the surgeon have each been more assisted by it than has the therapist. This is always the case, however, as the problems presented to him for solution are infinitely more complex and contain so many more indeterminate factors than those presented to the worker in any other branch of medical science. Therefore every well established therapeutical discovery should be hailed with delight, not only on account of the difficulty of its discovery, but more on account of the practical benefit conferred on society. I have been skeptical as to the benefits of electricity heretofore, on account of the confusion of terms and contradictory experience of those who have used it. I have also been disgusted to see medical fledgelings who were hazy upon the elementary principles, rush to secure an intricate electrical battery before they owned a U. S. dispensatory, and in fact before an authority of recognized standing would dare to put a prescription for electricity into writing. Much experimentation, however, has produced some knowledge. We at least know that some currents are stimulant, some sedative and some tonic. We know the dosage and some of the contraindications. We also know of some positive therapeutic uses.

The book before us is a large volume, and probably contains all that is known upon the subject. The author has labored to simplify the subject so that it can be understood and applied by those who have not studied it technically. Even the terms have been so simplified, and diseased conditions have been treated of from the clinical standpoint. The work opens with a short but comprehensive chapter on electro-physics. Then the physics of the galvanic currents are separately discussed, and their effects upon living tissues. The Static and Faradic currents are then discussed in the same manner. The various diseased conditions are then taken up and discussed in separate chapters. The work is essentially clinical and practical and is highly commended to all who may need a treatise on this subject. I. J. J.

